# Association of natural resistance-associated macrophage protein 1 polymorphisms with *Salmonella* fecal shedding and hematological traits in pigs

**DOI:** 10.14202/vetworld.2022.2738-2743

**Published:** 2022-11-29

**Authors:** Nattariga Suwannawong, Uschara Thumarat, Pitchayanipa Phongphanich

**Affiliations:** 1 Animal Production Innovation and Management Division, Faculty of Natural Resources, Prince of Songkla University, Songkhla 90112, Thailand; 2 Center of Excellence on Agricultural Biotechnology (AG-BIO/MHESI), Bangkok 10900, Thailand; 3 Department of Industrial Biotechnology, Faculty of Agro-Industry, Prince of Songkla University, Songkhla 90112, Thailand

**Keywords:** hematological trait, *Nramp1*, *Salmonella* shedding pig

## Abstract

**Background and Aim:**

Natural resistance-associated macrophage protein 1 encoding gene (*Nramp1*) plays a role in immune response and disease resistance. This study aimed to investigate the polymorphisms of *Nramp1* intron 6 concerning *Salmonella* shedding and hematological traits in pigs

**Materials and Methods:**

A total of 40 commercial pigs (three-way Large White x Landrace x Duroc cross) were genotyped using the polymerase chain reaction-restriction fragment length polymorphism (PCR-RFLP) method and analyze the relationship between the polymorphisms of the *Nramp1* gene and *Salmonella* fecal shedding and hematological parameters.

**Results:**

*Nramp1* was shown to be polymorphic in these pigs. The *Nramp1* gene has two alleles (A and B) and two genotypes (AB and BB). The BB genotype had a higher frequency than the AB genotype. A significant relationship between the BB genotype and the number of *Salmonella* in feces compared to the AB genotype (p < 0.05) on 7 days post-inoculation (DPI) was revealed in the association analysis. The single-nucleotide polymorphism at intron 6 in the *Nramp1* gene was linked to white and red blood cells 2 and 7 DPI (p < 0.05).

**Conclusion:**

The *Nramp1* gene was suggested by these findings to be potentially used as a molecular marker for the genetic selection of disease susceptibility in pig breeding.

## Introduction

Serious health problems, such as loss of appetite, depression, cough, high temperature, pneumonia, and septicemia, contribute to significant economic losses and pose a threat to human health, are potentially caused by *Salmonella* infection (salmonellosis) in pigs [1]. A decline in maternal antibody levels at the nursery stage is correlated with a high risk of infection. This can lead to the spread of salmonellosis to the finishing stage of production and, eventually, contamination of carcasses, with further adverse effects on human health [2, 3].

Improving resistance against *Salmonella* infectious disease traits has recently emerged as a critical goal in modern pig breeding programs. The pathogen response heritability has yet to be estimated due to difficulties in measuring phenotypes and defining what phenotype is relative and the difficulty of improving *Salmonella* infectious disease traits through traditional breeding methods. Molecular genetic methods, also known as marker-assisted selection (MAS), are ideal for improving *Salmonella* infectious disease traits in pigs. This is because MAS is more efficient, effective, and more dependable than phenotypic selection [4]. However, there is a paucity of published research regarding the genotyping of *Salmonella* susceptibility in pigs. *Salmonella* shedding in pigs was also previously linked to variations in the chaperone protein gene chaperonin containing TCP1 subunit 7 [5]. In addition, variations in porcine *TLR4* have been linked to the severity and duration of *Salmonella* Typhimurium shedding [6]. Recently, it was discovered that single nucleotide variations in the C-type lectin MBL1 and a single nucleotide variation in the cytosolic pattern recognition receptor NOD1 had been linked to an increased risk of internal colonization evaluated at slaughter and on-farm shedding [7]. It has been found in the previous research that the *Slc11a1* (solute carrier family 11 members 1, also known as natural resistance-associated macrophage protein [*Nramp1*]) gene, is involved in the susceptibility to pathogen infections in farm animals and has causal mutations [8]. The *Nramp1* gene is located on chromosome 15 q23–26 in pigs and has a 15-kilobyte length, with 15 exons and 14 introns [9, 10]. The polymorphism of the *Nramp1* gene has been related to immunological function [11–13], bacterial count [14], diarrhea in pigs [15, 16], and post-weaning piglet survivability [11]. One way to use genetics to increase resistance is to correlate them with immunological or hematological characteristics (e.g., cytokine production, leukocyte proliferation, packed cell volume (PCV), red blood cell (RBC) count, and serum levels of immunoglobulin) and the level of fecal *Salmonella* shedding [11–16].

Therefore, this study aimed to investigate the genetic variation of single-nucleotide polymorphism (SNP) genotyping in intron 6 of the *Nramp1* gene and its relationship to *Salmonella* shedding and hematological parameters in pigs.

## Materials and Methods

### Ethical approval

This study was approved by the Animal Ethics Committee of Prince of Songkla University (PSU) (record no. 2563-09-027).

### Study period and location

The study was conducted from January 2019 to July 2020 at the Animal Production Innovation and Management Division, Faculty of Natural Resources, PSU, Hat Yai Campus.

### Animals and sample collection

A total of 40 commercial pigs (three-way Large White × Landrace × Duroc cross) were selected from sows that tested negative for *Salmonella* spp. in their fecal samples. All pigs were −35 days of age, kept in mixed sex, raised under fully enclosed isolation facilities, and under identical management conditions at the Faculty of Natural Resources farm, PSU. The pigs with *Salmonella*-negative fecal samples were challenged with *S*. Typhimurium through oral administration with 2 mL of 1 × 10^9^ colony-forming units (CFUs). Blood samples (10 mL) were obtained from the jugular vein of each pig and immediately injected into tubes containing 5% ethylenediaminetetraacetic acid anti-coagulant for the measurement of hematological parameters before *Salmonella* inoculation (day 0) and after *Salmonella* inoculation (at 2, 7, 14, and 21 days post-inoculation [DPI]), respectively. Blood samples were stored at 4°C and analyzed within 24 h. Red blood cell and white blood cell (WBC) counts were determined using a hemocytometer [17]. Packed cell volume (PCV; %) was measured from microhematocrit tubes (NRIS microhematocrit tube, Herlev, Denmark) after centrifuging for 5 min at 10,000 rpm (8944 × *g*) in a microhematocrit centrifuge and the assistance of a hematocrit reader was used to determine hematocrit values (Nüve, NT 715, Ankara, Turkey). Giemsa-stained blood film was used to determine the differential leukocyte counts, and 200 cells were counted and classified, and the absolute leukocyte counts were examined at 1000× magnification (Microscopy Zeiss Primo Star, Carl Zeiss Inc., Oberkochen 73447, Germany) [17].

### Enumeration of *Salmonella* in fecal samples

The method of enumeration of *Salmonella* in fecal samples was modified by a previous study [18]. Briefly, individual fecal samples from 40 commercial pigs (three-way Large White × Landrace × Duroc cross) at 2, 7, 14, and 21 DPI were collected by rectal swab and serial-diluted 10 times with phosphate-buffered saline. *Salmonella* enumeration was performed on *Salmonella* differential agar (HiMedia, India). After that, the plates were incubated at 37°C for 24 h. Pink-red colonies on *Salmonella* differential agar plates were considered presumptive *Salmonella*. The presumptive *Salmonella* colonies (up to 10 per plate) were assessed by polymerase chain reaction (PCR) for the *invA* gene [19].

### DNA extraction

Genomic DNA was extracted from WBCs using the Thermo Scientific™ GeneJET Genomic DNA Purification Kit (Thermo Scientific, Waltham, MA, USA), and the concentrations of DNA samples were measured using NanoDrop Lite™ (Thermo Scientific, Waltham, Massachusetts, USA) (absorption at 260 and 280 nm and purity using the 260/280 ratio). Genomic DNA samples were adjusted to a final concentration of 20 ng/mL using TE buffer.

### Polymerase chain reaction and restriction fragment length polymorphism (PCR-RFLP)

Polymerase chain reaction was performed in a 10 μL mixture containing 1 μL genomic DNA (20 ng), 1 μL 10× PCR buffer, 1 μL 3 μM of primers for each candidate gene, 1 μL 1 mM of dNTP (Thermo Scientific), 0.8 μL 25 mM MgCl_2_, and 0.1 μL 5 U Taq DNA polymerase (Thermo Scientific). The *Nramp1* primer properties are forward: 5′-GCCAGCTTCCACAGTCTCCAG-3′; reverse: 5′-GGGGGTACAAAGGGGAAGAAG-3′ [11], and the amplified segment is about 483 bp in length. The PCR program was as follows: 95°C for 5 min, 30 cycles (94°C for 45 s, 58°C and 60°C for 30 s, and 72°C for 45 min). The final extension was at 72°C for 5 min. The PCR products were analyzed by electrophoresis on 2% agarose gel. For genotyping *Nramp1* genes, the PCR products were digested by *NdeI* enzymes. A total of 4 μL of each amplified DNA fragment was digested at 37°C for 15 min in a final volume of 10 μL, containing 1 U of the enzyme, 1 μL of restriction buffer, and 4.7 μL of sterile water. The genotype patterns were separated by 3% agarose gel electrophoresis and stained in GelStar (GelStar Inc., New York, USA). Agarose gels were visualized and photographed under the Aplegen Omega Lum G Gel Documentation System (Gel Company Inc., San Francisco, CA, USA).

### Statistical analysis

Genotype and allele frequencies were observed and the expected heterozygosity of the genotypes was obtained through the PCR-RFLP method. The Hardy-Weinberg equilibrium values were calculated using GenAlEx Version 6.51 [20]. Hematological data were analyzed using a one-way analysis of variance. Means were separated using Duncan’s multiple range tests. The *Nramp1* polymorphism and immune trait data were analyzed using the GLM procedure of SAS (SAS Institute, Cary, NC, USA). The following model was used: Y_ij_ = μ + S_i_ + G_j_ + e_ij,_ where Y_ij_ was the observation of *Salmonella* shedding, μ was the overall mean, S_i_ was the effect of sex, G_i_ was the effect of the SNP genotypes, and e_ij_ was the random residual effect.

## Results

### Polymorphism of the *Nramp1* gene

**Figure-1:**
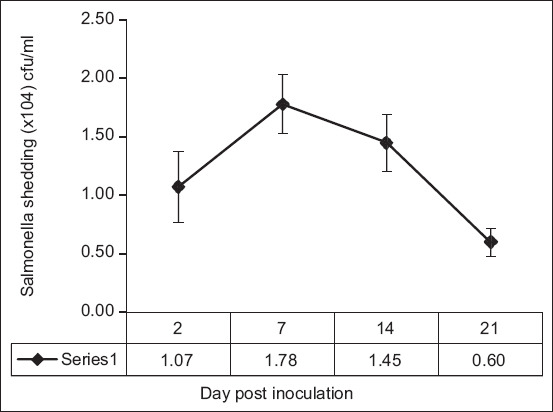
Fecal samples were collected from pigs orally infected with 1 × 10^9^ colony-forming unit (CFU) of *Salmonella* Typhimurium over time, measured by the CFU methods. Significant differences between day post salmonella infection (analysis of variance, error bars represent standard error of the mean p < 0.05).

In all pigs, the intron 6 of the *Nramp1* gene was genotyped using the PCR-RFLP technique (Figure-1). The restriction enzyme *Nde*I discovered that only two of the three genotypes for the *Nramp1* gene (AB and BB) were observed. The BB genotype had two fragments (373 bp and 110 bp), while the heterozygote AB genotype had three fragments (483 bp, 373 bp, and 110 bp, respectively). The genotype and allele frequencies of intron 6 of *Nramp1* in these pigs are shown in Table-1. The BB genotype was found to have a higher frequency than the AB genotype, and allele A had a substantially lower frequency than allele B. These pigs were demonstrated by this finding to have deviated significantly from the Hardy-Weinberg equilibrium (p < 0.05).

**Table-1:** Genotypes and allele frequencies of PCR-RFLPs of the *Nramp1* gene in pigs.

Genotype	n	Frequency	Allele	Frequency	χ^2^	p-value
AB	16	0.4	A	0.194	0.499	<0.05
BB	24	0.6	B	0.806	0.312	<0.05

Χ^2^ (HWE)=Chi-square test for Hardy-Weinberg equilibrium. PCR-RFLPs=Polymerase chain reaction and restriction fragment length polymorphism

### Association of the *Nramp1* gene with *Salmonella* fecal shedding

**Figure-2:**
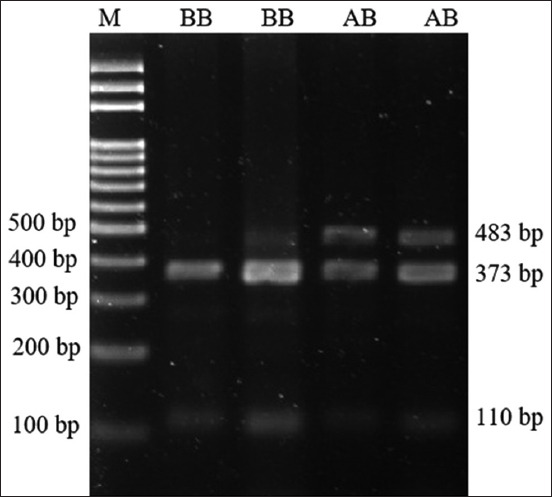
Polymerase chain reaction and restriction fragment length polymorphism digestion of intron 6 of the Nramp1 gene in pig. The lane M is Marker 100 bp ladder; the lane 2-3 are BB genotype, and 4–5 are AB heterozygote.

All pigs were negative for *Salmonella* at 0 DPI, with 100% of pigs shedding *Salmonella* at the second DPI. Pigs colonized with *Salmonella* are usually asymptomatic with varying severity and duration of fecal shedding. *Salmonella* shedding count in the pig feces was detected post challenge and revealed that the peak shedding occurred at 7 DPI and was significantly greater than on any other day (p < 0.05). The mean amount of *Salmonella* shed in the feces peaked at 1.78 (±0.25) × 10^4^ CFU/mL. *Salmonella* shedding in the feces was reduced in 14 and 21 DPI challenges (Figure-2). *Salmonella* shedding was not significantly affected by sex (data not shown). The effects of genotype on *Salmonella* shedding traits for the *Nramp1* gene are shown in Table-2. On 7 DPI, the pigs with the BB genotype shed significantly higher numbers of *Salmonella* in their feces than the AB genotype (p < 0.05).

**Table-2:** Association of SNP in intron 6 of the *Nramp1* gene with Salmonella fecal shedding ([×10^4^] CFU/mL [least square mean ± standard errors]).

Day post inoculation	*Nramp1* genotypes		p-value

	BB	AB	
2	0.78 ± 048	1.27 ± 0.39	0.43
7	2.41 ± 0.37^a^	1.38 ± 0.31^b^	0.04
14	1.95 ± 0.37	1.10 ± 0.31	0.08
21	0.45 ± 0.19	0.67 ± 0.16	0.38

^a,b^Least squares means within the row with different superscripts differ (p < 0.05). SNP=Single-nucleotide polymorphism, CFU=Colony-forming unit

### Association of the *Nramp1* gene with hematological traits

The effects of genotype on hematological traits for the *Nramp1* gene are shown in Table-3. At 2 DPI, the hematocrit was significantly higher for the AB genotype than for the BB genotype (p < 0.05). The RBC count in these pigs with AB and BB genotypes was not significantly different (p > 0.05). The WBC count and monocyte cell count were significantly lower for pigs with the AB genotype than for the BB genotype (p < 0.05) at 2 and 7 DPI. At the same time, neutrophil cell counts were significantly lower for pigs with the AB genotype than for the BB genotype at 2 and 14 DPI.

**Table-3:** Effects of intron 6 of *Nramp1* gene on the hematological trait in pigs (Least square means ± standard errors).

Hematological	Genotype	Day post inoculation			

		2	7	14	21
Hematocrit (%)	BB	34.80 ± 1.88^b^	46.30 ± 1.17	47.13 ± 1.64	42.50 ± 1.26
	AB	45.60 ± 1.51^a^	45.87 ± 0.94	45.02 ± 1.32	45.70 ± 1.02
RBC (×10^6^) (cell/mL)	BB	5.15 ± 0.48	5.87 ± 0.26	6.19 ± 0.39	6.26 ± 0.36
	AB	6.03 ± 0.38	5.89 ± 0.20	6.10 ± 0.31	6.00 ± 0.29
WBC (×10^3^) (cell/mL)	BB	39.70 ± 3.42^a^	33.70 ± 2.37^a^	26.17 ± 2.05	20.64 ± 1.82
	AB	24.33 ± 2.85^b^	23.09 ± 1.98^b^	28.66 ± 1.71	21.55 ± 1.54
Monocyte (%)	BB	14.62 ± 1.44^a^	7.15 ± 0.66^a^	5.13 ± 0.49	3.31 ± 0.36^b^
	AB	7.21 ± 1.20^b^	5.36 ± 0.55^b^	5.34 ± 0.41	4.32 ± 0.30^a^
Neutrophil (%)	BB	43.20 ± 1.76^a^	46.45 ± 2.58	47.17 ± 2.96^a^	42.15 ± 3.11
	AB	30.85 ± 2.41^b^	42.89 ± 1.89	37.26 ± 2.16^b^	38.24 ± 2.27

^a,b^Least squares means within a column and parameter with different superscripts differ (p < 0.05). RBC=Red blood cell, WBC=White blood cell

## Discussion

### Polymorphism of the *Nramp1* gene

In this study, we used PCR-RFLP methods to detect the sixth intron of the *Nramp1* gene polymorphism linked with *Salmonella* shedding and hematological response in nursery pigs to assess susceptibility to *Salmonella* in commercial pigs. Wu *et al*. [21] investigated the polymorphism of the sixth intron of the *Nramp1* gene in 11 sino-foreign swine. They discovered that the polymorphism was caused by a CA to TG mutation at 278–279 base sites of the *Nramp1* gene, resulting in *Nde*I failure to reorganize restriction enzyme sites. The AA genotype was not detectable, according to the findings of the current study. Likewise, it has been reported that the frequency of genotype AA in variants in the sixth intron of the *Nramp1* gene is very low, and it was not even detected in some pig populations [11, 16]. However, Chen *et al*. [15] revealed that three genotypes (AA, BB, and AB) were detected in Bamei and Large White breeds, and two genotypes (AA and AB) were detected in Landrace and Duroc breeds. Furthermore, we discovered three genotypes in Large White, Landrace, and Thai native pigs in a previous study [22]. As a result, some genotypes were eliminated for some traits due to a lack of balance between production and disease traits, selection intensity, and time among pig breeds.

The *Nramp1* gene was a candidate gene to control resistance and susceptibility to *Salmonella* spp. in mice [23] and chickens [24]. Cunrath and Bumann [25] suggested that the *Nramp1* protein plays a role in pathogenic infection resistance through a process involving magnesium ion transport, which provides host protection against various intracellular pathogens, such as *Salmonella* spp. In addition, mice with the *Nramp1* wild-type allele extrude Mn^2+^ faster than mice with the *Nramp1* mutation [26]. Furthermore, after infection, the expression of the *Nramp1* and *Nramp2 g*enes was modified in tissues, such as the liver, spleen, and caecum, all known sites of *S*. Typhimurium replication in chickens [27]. It is possible to infer the specific roles of the *Nramp1* and *Nramp2* genes in *S*. Typhimurium-induced illness from their varied f patterns in different organs and at different times following per os infection [27].

In this study, the mutation sites were at the sixth intron of the *Nramp1* gene, which is unclearly occurring within the splice junctions. However, if it is located within an intron junction or branch point site, or if it activates a cryptic splice site, it can still modify the splicing phenotype [11]. Furthermore, if it is an intronic microRNA, mutations may affect the gene expression homeostatic regulation system [28].

Hematological parameters in animals, including RBC and WBC counts, PCV, and hemoglobin concentration, are good indicators of physiological status and contribute to disease diagnosis and monitoring. Thus, they could be helpful in the selection of animals with genetic resistance to certain diseases [11–16]. There was no effect of sex on hematological traits (data not shown). The mean PCV between BB and AB genotypes was significantly different at 2 DPI, but they were both within the normal ranges of 22–55 [29] and did not cause anemia by analyzing hematologic parameters at 2, 7, 14, and 21 DPI. In 180-day-old body weight pigs, Wu *et al*. [11] found that the SNP in the sixth intron of the *Nramp11* gene was related to polymorphonuclear leukocyte levels and cytotoxin on monocytes. Moreover, the SNP in the one intron of the *Nramp1* gene was significantly associated with monocytes, rate of cytotoxin in monocytes, and CD4/CD8 T lymphocyte subpopulations in the blood [12]. Neutrophils and inflammatory monocytes are recruited during the early immune response to *Salmonella* in Peyer’s patches (PP) and mesenteric lymph nodes. This response is crucial for preventing the spread of germs to systemic organs [30]. Inbred mouse strains are resistant to *S*. Typhimurium infection, which is associated with increased leukocyte counts in the circulation and enhanced neutrophil influx into the peritoneum during infection [31]. However, pigs with different shedding outcomes developed distinct immune responses within the first 2 DPI with *S*. Typhimurium [32]. Moreover, a significant association between *Salmonella* shedding in their feces and higher numbers of circulating neutrophils, WBCs, and monocytes was shown by the variation in the sixth intron of the *Nramp1* gene. Thus, our study may imply that the BB genotype enhances the activity of monocytes by mediating iron homeostasis.

## Conclusion

In this study, we focused on the relationship between the intron6 of *Nramp1* gene polymorphism in pigs, hematological traits, and *Salmonella* shedding. According to our findings, the genetic mutation of intron 6 of the *Nramp1* gene affects hematological traits and *Salmonella* shedding. Pigs with the *Nramp1* BB genotype may be even more susceptible to shedding higher numbers of *Salmonella* than pigs with the AB genotype. Still, pigs with the *Nramp1* AB genotype were significantly associated with neutrophils, WBCs, and monocyte counts. The findings also revealed that the *Nramp1* gene could be used as a genetic marker in swine disease-resistance breeding for these *Salmonella* infection features.

## Authors’ Contributions

PP: Designed the study. PP, UT, and NS: Collected samples. UT and NS: Bacterial culture and microbial enumeration. PP and NS: Genotyping and data analysis. PP and UT: Drafted the manuscript. All authors have read and approved the final manuscript.
